# Demonstration of the Pfeiffer-Widal Typhoid-Serum Reaction

**Published:** 1897-09

**Authors:** John C. Spencer

**Affiliations:** San Francisco


### DEMONSTRATION OF THE PFEIFFER-WIDAL TYPHOID-SERUM REACTION.

BY JOHN C. SPENCER, M. D., SAN FRANCISCO.

(Read before the Medical Society of the State of California, April, 1897.)

In 1895, Widal, of Paris, continuing along the line of experiments by Gruber, Pfeiffer and Kolle, applied the knowledge so derived for the establishment of a reliable means of diagnosing typhoid fever in its early stages. The diagnostic value depends upon the presence in the blood and certain secretions, of an albuminoid body, by some called "agglutinin," by others "paralysin." This body in solution, brought in contact with a culture of typhoid bacilli, will, according to the degree of concentration, and also modified by the virulence of the particular colony of the bacilli, be followed by certain phenomena. The very active movements of typhoid bacilli will at first be slowed. Certain individuals will cease to move entirely, and many others will have grouped themselves into relatively large or small masses, suggesting somewhat the tangle which angle-worms are wont to form when lying in a bait-box. It is this grouping or clumping which constitutes the characteristic upon which the diagnostic value of the phenomenon rests. It has been observed that a strong admixture of normal serum, to a weak variety of the Eberth bacilli will be followed by clumping. When, however, a moderately weak dilution of the serum, or emulsion of dried blood from the suspected case is used, the reaction is positive.

The reaction has been found in using diabetic blood, placental blood, and from the blood of certain animals. When a case has been examined in the earlier stages with a negative result, and clinical manifestations persist, subsequent tests should be made. The further away from onset of clinical manifestations that a negative result is obtained, the stronger the presumption that we are dealing with a non-typhoid infection.

The earliest date upon which this reaction has been obtained, as far as I have been able to ascertain from reference to the already formidable accumulation of literature on the subject, has been on the third day (Appel and Thornbury, *Jour. of Am. Med. Ass'n.*, Vol. XXVIII., No. 6). The length of time that this agglutinative property remains in the blood has been variously observed as seven years, seventeen and one-half years, and thirty-one years.

At the outset, it was considered necessary to procure a few cubic centimeters of the blood of the patient from which the serum was obtained. Johnson, of Montreal, experimented with a drop of dried blood which was subsequently rubbed up with a few drops of sterilized distilled water. Likewise, instead of using a bouillon-culture of the Eberth bacilli, an emulsion was made of an agar-culture, using a very small quantity from the colony, and sterilized distilled water as before In the latter case it is very easy to get an artificial reaction because of the possible presence of too many bacilli. In any event a bouillon-culture is so easily made that it is always preferable, both because of the fewer bacilli present in a given quantity as well as the increase in virulence from such a fresh growth. The nature of these agglutinating bodies is essentially albuminoid. They have been assigned by Widal and others to the globulins.

Already nearly fifty separate contributions on the subject of this demonstration have appeared in the literature, in various languages. Doubtless most of my hearers have become familar with the few meager details which I have presented. For the reason that the subject has been so thoroughly and elaborately presented by others, I have foreborne to touch upon any but the essential facts. The far-reaching importance of the application of this principle can hardly be overestimated. Experiments on several hundred cases have established the reliability and certainty of this test. Its value as a diagnostic aid is immeasurable. Whereas, previous to this era, the most reliable means of diagnosing typhoid lay in the use of the Elsner culture-medium, whereby a delay of many days was necessary until the bacilli should have appeared in the stools, now the collection of a drop of blood from the lobe of the ear of the individual, caught on a cover-glass, a bit of mica or clean tissue-paper, enables the diagnosis to be established at as early a period as three days after the onset of the clinical manifestations, as we have seen. The inference most naturally drawn from the action of these agglutinating substances outside of the animal body, is the application of the same, either as such, or in a somewhat modified form, as a prophylactic or as an abortive after the typhoidal infection has once been established. This has already been the subject of experiment by two English observers, Wright and Semple (*British Medical Journal*, Jan. 30, 1897.)

If applicable in the case of typhoid, it would seem as though some similar method would be evolved for the prevention and cure of all of the acute infectious diseases. From my humble standpoint, it would seem as though medical science were but on the threshold of wondrous developments in the realm of preventive treatment, and more simple, certain, and rapid methods of diagnosis. Unless there should be a sudden and radical reversal of hitherto accepted facts and methods of procedure, the evolution will in all probability take place along these lines.—*Pacific Medical Journal.*

